# Open necrosectomy is feasible as a last resort in selected cases with infected pancreatic necrosis: a case series and systematic literature review

**DOI:** 10.1186/s13017-020-00326-z

**Published:** 2020-07-29

**Authors:** Lucia Ilaria Sgaramella, Angela Gurrado, Alessandro Pasculli, Francesco Paolo Prete, Fausto Catena, Mario Testini

**Affiliations:** 1 Unit of General Surgery “V. Bonomo”, Department of Biomedical Sciences and Human Oncology, University of Bari “A. Moro”, Polyclinic of Bari, Piazza Giulio Cesare, 11, 70124 Bari, Italy; 2 Department of Emergency and Trauma Surgery, Parma University Hospital, Viale Antonio Gramsci, 14, 43126 Parma, Italy

**Keywords:** Infected pancreatic necrosis, Pancreatic collection, Walled-off necrosis, Open surgical necrosectomy, Pancreatic abscess

## Abstract

**Background:**

Acute pancreatitis is a common inflammatory pancreatic disorder, often caused by gallstone disease and frequently requiring hospitalization.

In 80% of cases, a rapid and favourable outcome is described, while a necrosis of pancreatic parenchyma or extra-pancreatic tissues is reported in 10–20% of patients. The onset of pancreatic necrosis determines a significant increase of early organ failure rate and death that has higher incidence if infection of pancreatic necrosis (IPN) or extra-pancreatic collections occur.

IPN always requires an invasive intervention, and, in the last decade, the advent of minimally invasive techniques has gradually replaced the employment of the open traditional approach.

We report a series of three severe cases of IPN managed with primary open necrosectomy (ON) and a systematic review of the literature, in order to understand if emergency surgery still has a role in the current clinical practice.

**Methods:**

From January 2010 to January 2020, 3 cases of IPN were treated in our Academic Department of General and Emergency Surgery. We performed a PubMed MEDLINE search on the ON of IPN, selecting 20 from 654 articles for review.

**Results:**

The 3 cases were male patients with a mean age of 61.3 years. All patients referred to our service complaining an evolving severe clinical condition evocating a sepsis due to IPN. CT scan was the main diagnostic tool. Patients were initially conservatively managed. In consideration of clinical worsening conditions, and at the failure of conservative and minimal invasive treatment, they were, finally, managed with emergency ON. Patients reported no complications nor procedure-related sequelae in the follow-up period.

**Conclusion:**

The ON is confirmed to be the last resort, useful in selected severe cases, with a defined timing and in case of proven non-feasibility and no advantage of other minimally invasive approaches.

## Background

Acute pancreatitis (AP) is an inflammatory pancreatic disorder and the most common gastro-intestinal disease requiring hospitalization [1]. The incidence trend varies between 4.9 and 73.4 cases per 100,000 worldwide, and an increasing rate has been reported [2–6].

Gallstones are the leading cause of AP, accounting for 35–40% of cases, characterized by abdominal pain and elevation of pancreatic enzymes in the blood. In most of cases (80% of patients), a rapid and favourable outcome is described. However, approximately 10–20% [3] of patients develop necrosis of pancreatic parenchyma or extra-pancreatic tissues determining a significant increase of early organ failure rate (38%) and death (15%) [7, 8]. Patients with severe complications of acute necrotic pancreatitis (ANP) or peripancreatic fluid collection are currently treated with a primary conservative approach, and interventions are avoided or postponed until the necrosis becomes walled-off (WON) and liquefied. The major cause of death and multiorgan failure in case of complicated pancreatitis is the infection of pancreatic necrosis (infected pancreatic necrosis IPN) or extra-pancreatic collections that develop in approximately 30% of patients and lead to an increase of mortality to approximately 39% [9–14]. In such cases (about one third), an invasive intervention becomes mandatory to avoid a life-threatening evolution [9, 15].

The traditional approach to the treatment of IPN had historically been open necrosectomy (ON) with the complete removal of the infected tissue. Nevertheless, due to the still high rates of complications and death characterizing the ON, in the last decade, it has been gradually replaced by minimally invasive procedures that seem to reduce the incidence of postoperative new-onset organ failure compared with the open procedures [12, 13, 16–18]. Less invasive procedures, indeed, have been successfully proposed and tested, such as percutaneous drainage (PD), endoscopic transgastric necrosectomy (ETN), or video-assisted retroperitoneal debridement (VARD) [19, 20]. Recent evidence suggests that the surgical “step-up approach”, including PD, followed, if necessary, by minimally invasive necrosectomy, could be preferred to open surgery and be considered as the standard treatment for IPN with lower rates of postoperative adverse events (40%) and long-term morbidity [14]. Only few randomized controlled trials (RCT) comparing the step-up approach with ON have been performed, and the high variability of surgical procedures and materials proposed in the step-up procedures justifies the difficulty in the technique standardization [21–24]. Therefore, ON with repeated laparotomies is considered the last choice, whereas other therapeutic options have failed.

The aim of this study was to report our institution experience over the last 10 years and to review the recent literature on the open surgical management of IPN in order to understand if traditional surgical necrosectomy still has a role in the current clinical practice.

## Main text

### Cases presentation

From 2010 to 2020, 3 cases of IPN have been treated in our Academic Department of General and Emergency Surgery. We retrospectively reviewed clinical presentation, diagnosis, treatment, and follow-up.

#### Case 1

**Fig. 1:**
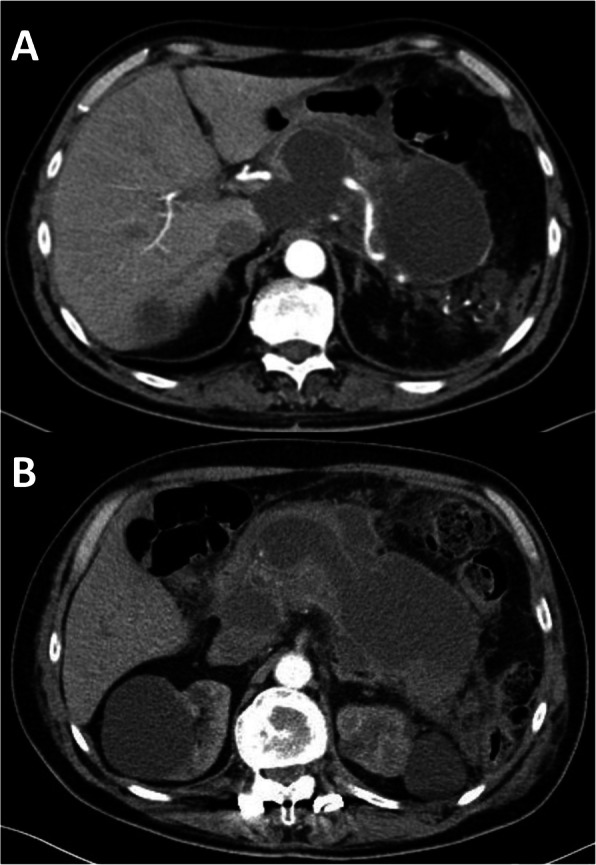
Case 1: Preoperative CT scan. **a**, **b** CT scan of the abdomen and pelvis revealing a heterogeneous 85 × 68 × 60-mm pancreatic necrotic collection

A 65-year-old man was admitted to our Department of General Surgery after 15 days from the onset of severe acute gallstone pancreatitis medically treated in another hospital. His past medical history revealed essential hypertension under treatment and splenectomy for trauma. A CT scan performed 4 days prior to our hospitalization revealed multiple pancreatic and peri-pancreatic necrotic fluid collections involving the retro-duodenal space and the splenic lodge. At the admission, the patient was febrile (38.3 °C/100, 94 F) with other normal vital signs. He referred left upper quadrant abdominal pain, abdominal distension, anorexia, and loss of weight accompanied with constipation. At physical examination, the abdomen was generally distended but not tender, with a large palpable mass in the left upper quadrant. Routine biochemistry showed leucocytosis (11.27 × 10^3^/uL) and an increase in inflammatory markers (C-reactive protein: CRP 102.0 mg/L). A CT scan of the abdomen and pelvis revealed a heterogeneous 85 × 68 × 60-mm pancreatic necrotic collection (Fig. 1a, b) with extension to the retro-duodenal space, to the mesenteric root, and to the splenic lodge, while an magnetic resonance cholangiopancreatography (MRCP) confirmed the presence of biliary endoluminal sludge in the gallbladder and terminal bile duct (BD) causing obstruction and proximal distension (10-mm diameter).

The patient was conservatively treated with fluid resuscitation, antibiotics, and supportive treatment as well as nutritional support. Moreover, an endoscopic retrograde cholangiopancreatography (ERCP) with endoscopic sphincterotomy and clearing of BD was performed. Because of the worsening of clinical condition, a percutaneous US-guided drainage was attempted, failing in relation to the its heterogeneous and sepimented content with prevalence of solid necrotic tissue. With the occurrence of clinical worsening and hemodynamic instability, an emergency open surgical debridement was performed 35 days after ANP clinical onset. An extended adhesiolysis was performed, followed by cholecystectomy, opening of gastrocolic ligament with marsupialisation of the collection wall and ON with debridement of necrotic content. Finally, drainages were left in place. During the operation, the fluid component was aspirated and sent to lab for culture and sensitivity. Infection of WON, indeed, was confirmed by bacterial culture positive to *Enterococcus Faecium*.

The post-operative time was uneventful. Drainages were removed on the 15th post-operative day and after 17 days, the patient was discharged. He has been followed up for a post-operative time of 8 months during which no sequelae were identified, and a follow-up CT scan showed that the infected WON had resolved with diminished inflammatory changes in the pre-pancreatic region.

#### Case 2

**Fig. 2:**
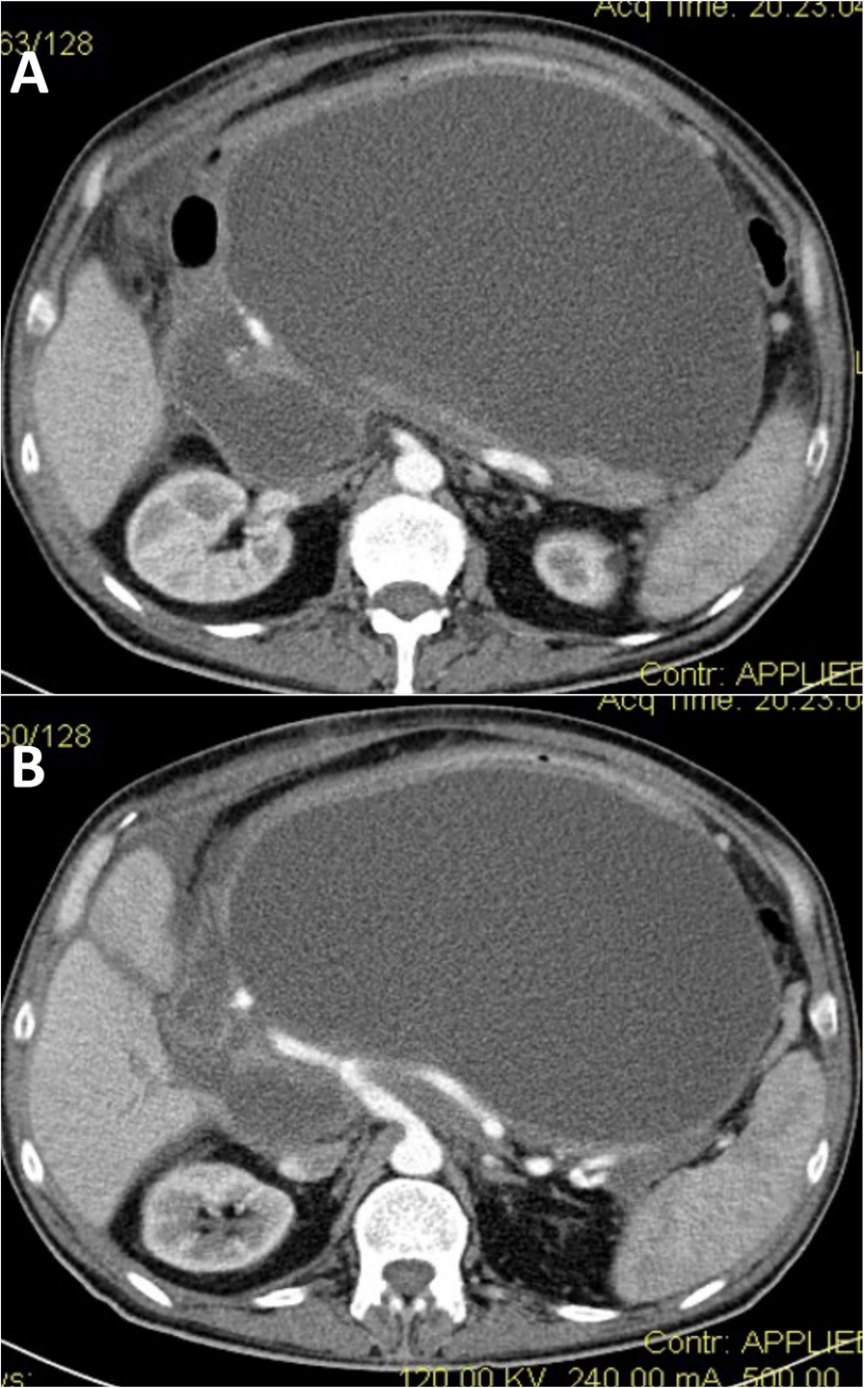
Case 2: Preoperative CT scan. **a**, **b** CT scan showing a heterogeneous and sepimented 23 × 15-cm low-density lesion suggestive for infected pancreatic necrosis

**Fig. 3:**
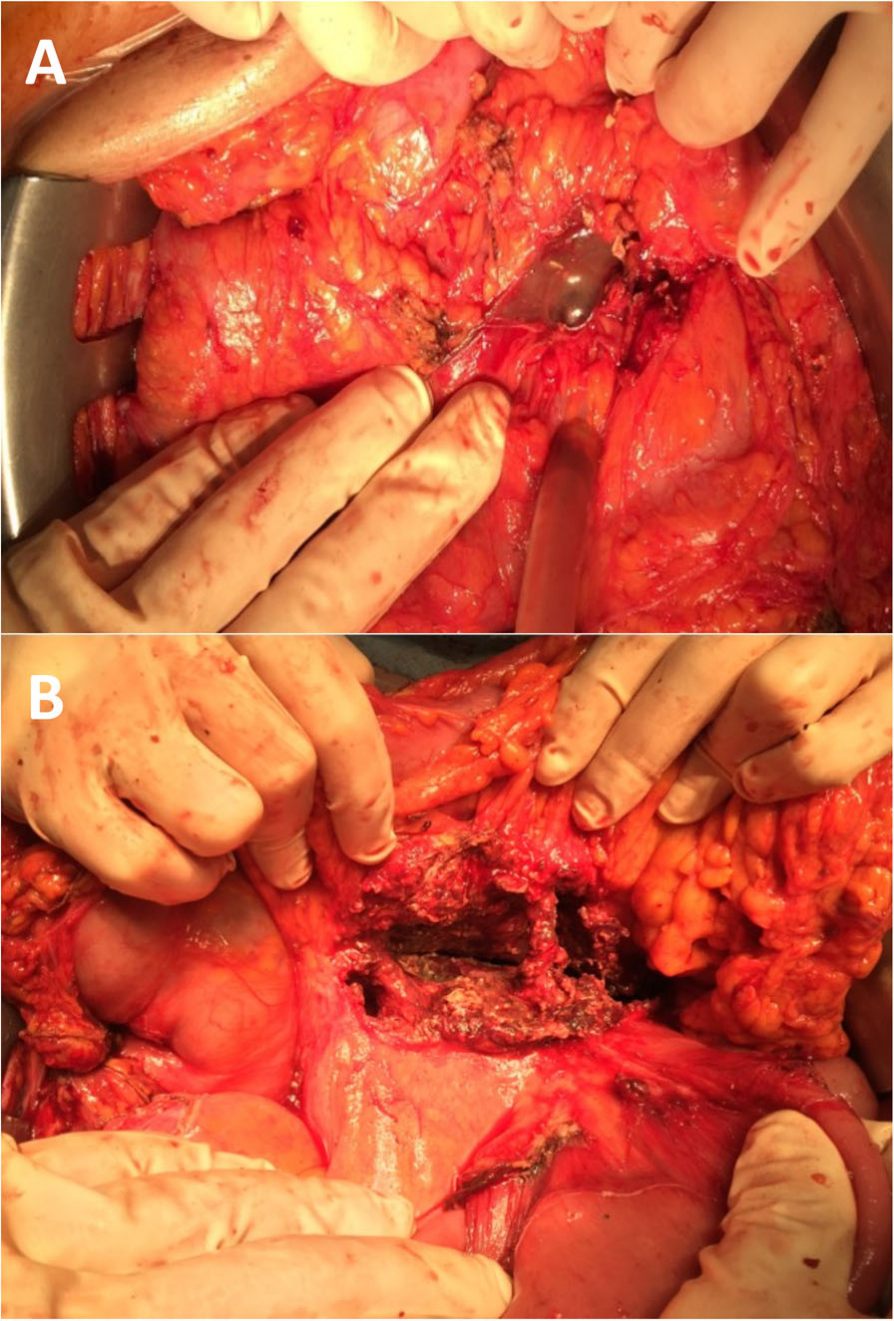
Case 2: Intraoperative images. **a**, **b** Intraoperative imaging of open necrosectomy

A 60-year-old man referred in emergency to our Academic Unit of General Surgery with a severe clinical condition evocating a sepsis due to IPN. At the admission, he was tachycardic (heart rate 120 beats/min) and febrile (38.7 °C/101.66 F), presenting abdominal pain and distension, general malaise, and anorexia. At physical examination, the abdomen was distended, and a large mass was palpable. He had been submitted to cholecystectomy 1 month before admission, and he had developed a severe post-operative ANP. Routine biochemistry evidenced an increase in inflammatory markers (PCR 171.0 mg/L) and leucocytosis (14.42 × 10^3^/uL), and CT scan and MRCP showed a heterogeneous and sepimented 23 × 15-cm low-density lesion, suggestive for infected WON with extension to the retro-duodenal space, the mesenteric root, and the splenic lodge surrounding splenic vessels (Fig. 2a, b). The collection also caused compression of the liver and hepatic hilum, stomach, and intestinal obstruction. He was managed with antibiotics therapy and fluid resuscitation. Nevertheless, the patient developed severe sepsis (leucocytosis 18.96 × 10^3^/uL; presepsin 538 pg/mL) evolving into shock, and the minimally invasive approach was waived in favor of emergency surgical debridement. On the 30th day after the onset of ANP, an extended adhesiolisis was performed with opening of gastrocolic ligament and of the WON, and the IPN was drained with an ON, necrotic debridement, and drainage placement (Fig. 3a, b).

The post-operative time was uneventful. Drainages were removed on the 13th postoperative day, and the patient was discharged on the 15th day.

#### Case 3

**Fig. 4:**
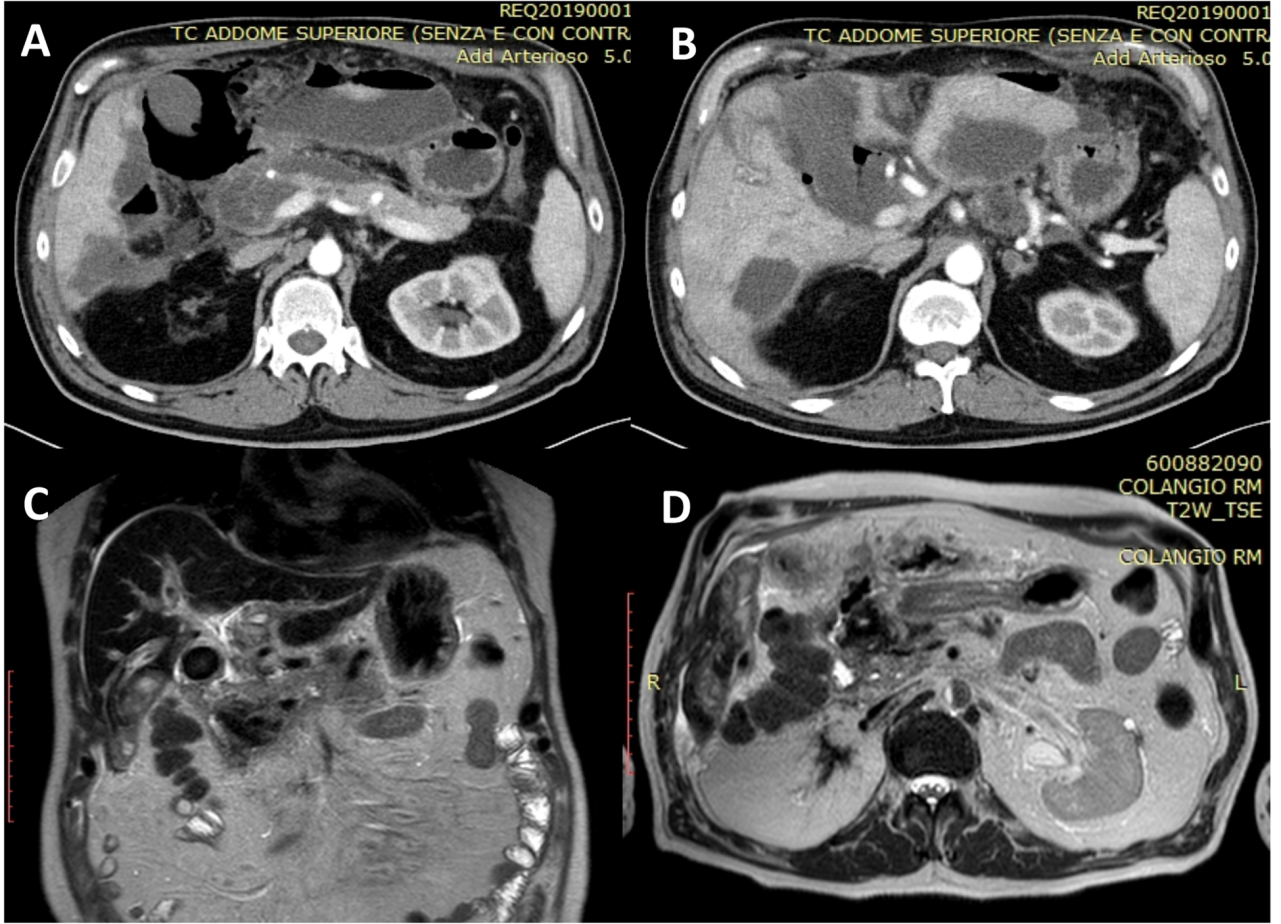
Case 3: Preoperative CT scan and MRCP. CT scan (**a**, **b**) and MRCP (**c**, **d**) showing acute gallstone cholecystitis and signs suggestive for infected pancreatic necrosis

A 59-year-old male was admitted from the emergency room for severe continuous epigastric pain that had originated a few hours previously at home. He had been discharged the day before after recovering from an episode of acute cholecystitis managed with conservative treatment and having been scheduled for elective cholecystectomy. He underwent an emergency CT scan confirming the gallstone disease and showing a new finding of 6-mm stone impaction at the distal end of the common BD. Blood tests were unremarkable. After 24 h, blood tests showed evidence of a marked elevation of serum amylase (975 U/L) and lipase (4395 U/L) without significant jaundice, and a new CT scan, performed 48 h after the previous one, showed the onset of ANP, with duodenal swelling. A mandatory oesophagogastroduodenoscopy (OGD) was then performed revealing patches of gastric antrum and duodenal necrosis. He was kept on conservative management with parenteral nutrition for more than 10 days before another OGD that showed a stabilization of the severe but not perforated duodenal disease. On hospital day 25, he developed pyrexia up to 38.5 °C (101.3 F) and showed high white cell count (21.06 × 10^3^/uL [normal range 3.70–9.70]). CT scan and MRCP showed acute gallstone cholecystitis and signs suggestive for IPN (Fig. 4a–d). Fluid collection in the right retroperitoneal and perihepatic space and severe oedema of peri-pancreatic adipose tissue were also documented, with abdominal and pelvic effusion.

Due to the compromised duodenal status, an ERCP was withheld, and we decided to perform a hazardous surgical exploration with a program of cholecystectomy and necrotic debridement in consideration of the sudden clinical worsening. After 1 month from the clinical onset of ANP, an open extensive adhesiolisis, cholecystectomy, and necrosectomy were performed, along with gastro-entero anastomosis to divert the alimentary tract away from the damaged duodenum; both the hepatic and the common BD appeared weakened and under dense adhesions, and it was too risky to position a biliary drainage, nor it was possible to consider a high-morbidity alternative as an emergency pancreaticoduodenectomy [25, 26]. The IPN was drained, and drains were left in place. On the 10th postoperative day, anaemia (Hg 7.8) with a CT evidence of self-limiting bleeding from epiploic vessels managed with embolization occurred. Moreover, a well-controlled and drained duodenal lateral fistula from the posterior wall of the weakened duodenum was evident, and it was initially conservatively managed, keeping the drain in place. Two months after the admission, the patient was discharged in fairly good clinical conditions, on a diet with oral intake. After 1 month of follow-up, an external internal drainage was positioned in order to approach the persistent duodenal fistula. The patient died after 3 months for a brain stroke.

### Review of literature

A systematic review was performed by searching in PubMed/MEDLINE from 2010 to 2020, using the following keywords: “infected walled off pancreatic necrosis” OR “open necrosectomy AND necrotizing pancreatitis” OR “Pancreatitis AND infected AND necrosis AND surgery”.

### Inclusion and exclusion criteria

All studies meeting the inclusion criteria were considered for the review: cohorts of almost 15 patients with necrotizing pancreatitis undergoing primary ON for suspected or confirmed IPN and in which primary outcomes were reported (percentage of infected, open surgery success rate, mortality, and complications). Comparative studies, concerning the employment of different techniques with a total cohort of patient higher than 15, were considered in case of almost 10 patients with IPN managed with primary ON.

Contrariwise, the following were excluded: non-English papers, studies with study cohorts of less than 15 patients, studies including patients with chronic pancreatitis or with results for acute pancreatitis not reported separately, studies including patients classified as pseudocysts or pancreatic abscess as defined by the 1992 Atlanta classification, studies including sterile pancreatic necrosis with results of IPN not reported separately, or studies concerning uncomplicated sterile necrotic pancreatitis.

### Study selection

**Fig. 5:**
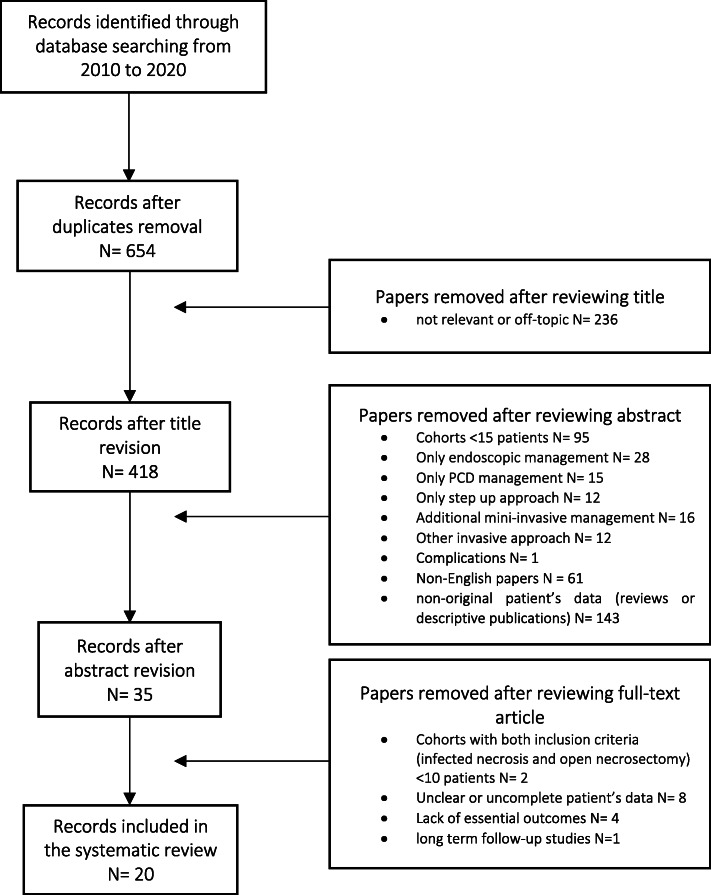
Study selection flowchart

The search returned 654 papers after removing duplicates. The study selection flowchart is shown in Fig. 5.

Six hundred and eighteen publications were excluded after reviewing title, abstract, and full text for the following reasons: non-English papers (*N* = 61); not relevant or off-topic (*N* = 236); studies with cohorts of less than 15 patients (*N* = 95) or with less than 10 patients managed with primary open surgery for IPN (*N* = 2); cohorts including only endoscopic management (*N* = 28) or only percutaneous management (*N* = 15); cohorts of only step-up approaches (*N* = 12), including any additional mini-invasive management (*N* = 16) or other invasive approaches (*N* = 12); cohorts that did not report essential outcomes (*N* = 4); and cohorts excluded because of non-original patient data (reviews or solely descriptive publications*, N* = 143; complications, *N* = 1). Furthermore, nine studies were also excluded because patient’s data were unclear or incomplete (*N* = 8) or because of long-term follow-up studies (*N* = 1).

Finally, a total of 20 studies were included in the systematic review [12, 14, 27–44].

### Study characteristics

This systematic review analyzes the results of patients submitted to primary ON for IPN. Studies and patients’ characteristics are summarized in Table 1.

**Table 1:** Patient’s characteristics

Study	Country	Year	Study design	Inclusion Criteria	N° open/tot	study period	M/F	Age (mean)	Aetiology (%)	APACHE II^a^	CT Severity^b^	ICU admission (%)	Comorbidities	Organ Failure at presentation (%)
Husu et al. [26]	Finland	2020	Retrospective cohort	Primary open surgical necrosectomy for pancreatic necrosis	109	2006–2017	96/13	52 (42–61)	A 62(56.9) B 25(22.9) I 11(10.1) Hyper trig 0 O 11(10.1)	Nr	Nr	44 (40.4)	CV 22 P 10 CRI 4 D 11 OT 5	12 (11.0)
Martinez et al. [27]	USA	2019	Rretrospective cohort	Primary open surgical necrosectomy for pancreatic necrosis	34/56	2007–2016	24/10	55.9 ± 14.4	A 4(11.8) B 16(47.1) I 0 Hyper trig 4(11.8) O 10(29.4)	Nr	Nr	Nr	Acute renal F 18 (52.9) Acute respiratory F 18 (52.9)	multiple 16 (47,1)
Cao et al. [28]	China	2019	Retrospective cohort	Primary open surgical necrosectomy for infected pancreatic necrosis	45	2015–2017	32/13	51.89 ± 12.92	A 6 (13.3) B 16 (35.6) H 20 (44.4) O 3 (6.7)	Nr	9 ± 1.1	Nr	CV 22 P 1 CRI 2 D 8 OT /	single 17 (37.8) multiple 7 (15.6)
Šileikis et al [29]	Lithuania	2017	Retrospective cohort	Primary open surgical necrosectomy for pancreatic necrosis	53/95	2007–2016	34/19	56.5 ± 16.1	Nr	Nr	7.6 ± 1.9	Nr	Nr	multiple 41 (77.4)
Wroński et al [30]	Poland	2017	Retrospective cohort	Primary open surgical necrosectomy for pancreatic necrosis	22/70	2007–2014	16/6	49 (36–61)	A 12(54.6) B 4 (18.2) O 6 (27.3)	Nr	8 (6–10)	Nr	Nr	4 (18.2)
Rash et al [31]	Germany	2016	Retrospective multicenter cohort	Primary open surgical necrosectomy in severe pancreatitis	30/220	2008–2014	2.3:1	55 (18 ± 82)	Nr	Nr	Nr	Nr	Nr	25 (73.3)
Gomatos et al [32]	UK	2016	Retrospective cohort	Primary open surgical necrosectomy for pancreatic necrosis	120	1997–2013	86/34	58.5 (42–70)	A 53 (44.2) B 37 (30.8) I 5 (4.2) O 25 (20.8)	8	7	36 (30)	Nr	multiple 30 (25)
Barreda et al. [33]	Perù	2015	Retrospective cohort	Primary open surgical necrosectomy in emphysematous necrotizing pancreatitis (gas within the pancreatic necrosis on CT)	36/56	2003–2011	nr	54 (16–80)	A 4 (7.1) B 46(82.1) I 3(5.4) Hyper trig 2 (3.6) O 1(1.8)	14 (6–28)	9.4 (8–10)	Nr	Nr	single 30 (83.3) multiple 21 (58.3)
Pupelis G et al [34]	Latvia	2014	Prospective cohort	Primary open surgical necrosectomy for infected pancreatic necrosis	39/70	2004–2014	32/7	47 (41–62)	A 23 (59) B 6(15.4) O 10 (25.6)	12 (7–18)	8 (6-10)	Nr	ASA III 31 (79.5%)	multiple 15 (38.5)
Tan V et al [35]	France	2014	Retrospective multicenter cohort	Primary open surgical necrosectomy for infected pancreatic necrosis	21/32	2005–2011	14/7	52(47–60)	A 6 (28.6) B 6(28.6) O 9 (42.8)	12 (10–16)	6 (5—6)	Nr	CV 14 D 5 OT 9	13 (60)
Madenci et al [36]	USA	2014	Retrospective cohort	Primary open surgical necrosectomy for pancreatic necrosis	68	2006–2009	48/20	54.2 ± 1.7	A 26 (38.2) B 19 (27.9) I 5(7.4) Hyper trig 5 (7.4) O 13(19.1)	10.9 ± .8	7.6 ± .3	22 (32.3)	Nr	24 (35.3)
Pascual et al [37]	Spain	2013	Retrospective cohort	Primary open surgical necrosectomy for infected pancreatic necrosis	21/39	1998–2010	10/11	62.5 ± 14.6	A 3 (14.3) B 14 (66.7) I 0 O 4(19)	10.6 ± 5.6 (r = 2–22)	10 (6–10)	Nr	11 (52.4 %)	single 3 (14.3) multiple 11(52.4)
Tu et al [38]	China	2012	Retrospective cohort	Primary open surgical necrosectomy for infected Pancreatic necrosis	32/50	2006–2012	19/23	48.7 (33–71)	A 3 (9.4) B 28 (87.5) Hyper trig 1 (3.1)	13.4 ± 5.2	5.3 ± 1.6	Nr	Hypovol 14 Hypox10 ARenalF3 Acute GH 1	Nr
Senthil Kumar et al [39]	India	2012	Retrospective cohort	Primary open surgical necrosectomy for suspected or confirmed infected necrosis	15/30	2008–2011	15/0	41 (30–55)	A 9 (60) B 5 (33.3) O 1 (6.7)	9 (5–20)	CTI 7 (3) CTI 8–10 (12)	11	Nr	single 8 (53.3) multiple 2 (13.3)
Bausch et al [40]	Germany	2012	Retrospective cohort	Primary open surgical necrosectomy for pancreatic necrosis	30/62	1998–2010	17/13	64 (25–88)	A 5 (16.7) B 4 (13.3) I 2 (6.7) O 19 (63.3)	Nr	Nr	Nr	Nr	multiple 22 (73.3)
Tan et al [41]	China	2012	Retrospective multicentre cohort	Primary open surgical necrosectomy for suspected or confirmed infected necrosis	51/76	2008–2010	33/18	44.1	A 24 (31.5) B 30 (39.5) I 4 (5.3) O 18 (23.7)	12.46–5.8	4.91–1.23	Nr	Nr	Nr
Doctor et al [42]	India	2011	Retrospective cohort	Primary open surgical necrosectomy for infected pancreatic necrosis	59/61	1998–2009	49/12	43(18–73)	A 14 (23) B 25 (41) I 3 (4.9) O 19 (31.1)	> 9	≥ 7	59	Nr	Nr
Boland et al [43]	USA	2010	Retrospective cohort	Primary open surgical necrosectomy for infected pancreatic necrosis	21	2002–2008	13/8	53 (28–86)	A 3 (14.3) B 8 (38.1) I 4 (19.0) O 6 (28.6)	Nr	Nr	Nr	CV 7 (%) ARI 2 (%) D 5 (%) OT 4.	9 (43)
Raraty et al [11]	UK	2010	Retrospective cohort	Primary open surgical necrosectomy for pancreatic necrosis	28	2000–2008	Nr	Nr	Nr	10.5 (5–26)	Nr	Nr	Nr	Nr
van Santvoort et al [13]	Netherlands	2010	Multicentre randomized study	Primary open surgical necrosectomy for suspected or confirmed infected necrosis	45/88	2005–2008	33/12	57.4 ± 2.0	A 5 (11) B 29 (64) O 11 (24)	15.0 ± 5.3	8 (4–10)	29 (64%)	CV 21 (47%) P 4 (9%) CRI 2 (4%) D 4 (9%) OT/. ASA III 14 (31)	single 22 (49) multiple 13 (29)

^a^Scores on the Acute Physiologic and Chronic Health Evaluation II (APACHE II) scale range from 0 to 71, with higher scores indicating more severe disease

^b^Scores on the CT severity index range from 0 to 10, with higher scores indicating more extensive pancreatic necrosis and peripancreatic fluid collections

*Nr* not reported, *A* alcoholic, *B* biliary, *I* iatrogenic, *Hyper trig* hypertriglyceridemia, *O* other

The vast majority of the analyzed studies (*N* = 18; 90%) were retrospective with the exception of 1 multicentre randomized study and 1 prospective study.

The pooled data of this review is 879 patients submitted to open surgery for suspected or preoperatively confirmed IPN. The mean number of patients involved *per* study is 43.9, ranging from 15 to 120; the majority of patients were men while the reported age ranges from 16 to 88.

Not all studies are complete in providing all predictive severity scores, preoperative clinical presentation, and details. Seventeen studies, indeed, have indicated disease aetiology, whose majority was alcoholic or biliary. The CTI score (CT severity index) is reported in fourteen studies with a value usually higher than 7; APACHE II score (Acute Physiologic and Chronic Health Evaluation II) is present in 12 studies with a value greater than 9, while details on preoperative organ failure and intensive care unit (ICU) admission are present in sixteen and six studies, respectively. Nine studies underline preoperative American Society of Anaesthesiologists (ASA) score and/or pre-existing comorbidities, with cardiovascular disease being the most commonly present followed by acute or chronic renal insufficiency and pulmonary dysfunction. Details on operative management and post-operative course are reported in Table 2. Nine studies have highlighted details on collection characteristics describing how a large part of patients has an extension of necrotic tissue involving up to 50% of parenchyma at the time of surgery.

**Table 2:** Data on operative management and post-operative course

Study	Indications to surgery	technical note	IPN (%)	Collection	Time until necrosectomy from admission in days	Pathogens (most frequently found %)	Mortality (%)	Re-necrosectomies	New onset organ failure OF (%)	PF (%)	POB (%)	NB (%)	PEI (%)	Follow-up month
Husu et al. [26]	*Infected pancreatic necrosis *Organ failure *Prolonged pain *Bleeding *Gastric outlet obstruction	Upper transvers subcostal laparotomy	85/109 (77.9)	Not assessable 45 (41.3%) < 30% 31 (28.4%) 30–50% 11 (10.1%) > 50% 22 (20.2%)	36 (22–59)	Nr	25 (22.9)	27 (24.7)	Nr	43 39.4	11 10.1	Nr	Nr	Nr
Martinez et al. [27]	*Infected pancreatic necrosis *Necrotic pancreatitis *Organ failure	Longitudinal midline trans-peritoneal approach	26/34 (76.4)	Nr	10.5 (4–23)	-Yeast 10 (29.4) -*Strept. Angin*. 9 (26.5) -*E. Coli* 7 (20.6)	7 (20.6)	11/34 (32.4)	Nr	Nr	Nr	Nr	Nr	Nr
Cao et al. [28]	*Infected pancreatic necrosis *Organ failure	*Trans-lesser sac approach 32 *Eetroperitoneal approach 11 *Combined 2	45 (100)	Ho 16 He 33	51.84 ± 28.48	Nr	4 (8.8)	Nr	Nr	10 22.2	2 4.4	3 6.7	1 2.2	18
Šileikis et al [29]	*Infected pancreatic necrosis FNA *Suspected Infected pancreatic necrosis/Organ failure/sepsis	Nr	42 (79.3)	Nr	28.8 ± 14.0	Nr	34 (64.1)	Nr	Nr	38 71.7		Nr	Nr	Nr
Wroński et al [30]	*Confirmed or suspected Infected pancreatic necrosis FNA 17 *Persisting unwellness 5	Transperitoneal necrosectomy -midline -subcostal	17 (77.3)	< 30% 5 (22.7%) 30–50% 4 (18.2%) > 50% 9 (40.9%)	40.5 (27–71)	Nr	6 (27.3)		12 (54.5)	7 31.8	6 28.6	Nr	Nr	Nr
Rash et al [31]	*Infected pancreatic necrosis FNA *Suspected Infected pancreatic necrosis/Organ failure/sepsis		25/30 (83.3)	Nr	> 10 days after onset of symptom	Nr	10 (33.3)	Median number of interventions 4 (r. 0–78)	Nr	Nr	Nr	10 33.3	Nr	Nr
Gomatos et al [32]	*Infected pancreatic necrosis *Organ failure/sepsis *Persisting unwellness	transperitoneal necrosectomy	78 (65)	Not assessable 20 (16.7%) < 30% 27 (22.5%) 30–50% 34 (28.3%) > 50% 39 (32.5%)	24 (12.8–42.3)	Nr	28 (23.3)		42 (35.0)	14 11.7	18 15.0	Nr	Nr	Nr
Barreda et al. [33]	*Infected pancreatic necrosis 31 *Organ failure 5	/	28/36 (77.7)	Nr	38.5 (13–90)	*E. coli* 13 (46)	6 (16.6)	Nr	nr	nr	nr	Nr	Nr	Nr
Pupelis G et al [34]	*Infected pancreatic necrosis *Organ failure	Longitudinal midline or bilateral subcostal trans-peritoneal approach	32 (82)	> 50% 22 (56.4%)	22 (17–27)	*E. coli* Enterococcus	5 (12.8)	Nr		5 12.8	9 23.1	Nr	Nr	Nr
Tan V et al [35]	*Infected pancreatic necrosis 21	Bi-subcostal trans-peritoneal approach (cholecystectomy associated) post-operative irrigation	19 (90)	Nr	21 (3–120)	Nr	3 (14)	Nr	5 (17)	8 38.0	3 14.0	4 19.0	4 19.0	16.1
Madenci et al [36]	*Infected pancreatic necrosis 43 *Failure to thrive 13 (19.1) *Sepsis syndrome 9 (13.2) *Biliary obstruction 2 (2.9) *Hemorrhage 1 (1.5)	Transmesocolic 47 (70.1%) Anterior 20 (30.3%) Retroperitoneal 3 (4.5%)	*Preop detected 43 (63) *Newly detected 11 (16.2)	Not assessable 7 (14.9%) < 30% 15 (31.9%) 30–50% 5 (10.6%) > 50% 20 (40.5%)	39.5 (29–73)	Polymicrobial 19 (28)	6 (8.8)	10 (14.7)	17 (25.0)	49 74.2	12 17.7	14 20.6	7 10.3	23
Pascual et al [37]	*Infected pancreatic necrosis 21	Open debridement, necrosectomy and drainage lavage	21/21 (100)	Nr	18.6 ± 16.9 (*r* = 1–76)	Nr	9 (42.9)	Nr	10 (47.7)	6 28.6	4 19.0	4/12	2/12	Nr
Tu et al [38]	*Infected pancreatic necrosis *Organ failure	Longitudinal or crosscut incision; trans-lesser sac approach	32/32 (100)	Nr	18.3 (6–31)	Nr	4 (12.5)	Nr	Nr	11	2	Nr	Nr	Nr
Senthil Kumar et al [39]	*Infected pancreatic necrosis 15	Transperitoneal necrosectomy	15/15 (100)	He 15	31 (17–45)	Nr	1 (6.7)	2	3	1	5	Nr	Nr	Nr
Bausch et al [40]	*Infected pancreatic necrosis 1 *Organ failure/sepsis 16 *Other 13	Transperitoneal necrosectomy	25/30 (100)	Nr	11 (0–77)	nr	19 (63.3)	22	Nr	5	8	Nr	Nr	Nr
Tan et al [41]	*Infected pancreatic necrosis 51	Transperitoneal necrosectomy	67/76 (88.6)	Nr	30 (13–46)	*Pseudomonas*.	3 (5.9%)	1	Nr	28	3	Nr	Nr	Nr
Doctor et al [42]	*Infected pancreatic necrosis *Organ failure/sepsis *Persisting unwellness	Transperitoneal necrosectomy	51 (83.6)	Nr	29 (13–46)	*E. coli, Klebsiella. Acinetobacter.*, *Pseudomonas*	6 (9.8)	2	Nr	31 (50.8)	4	Nr	Nr	Nr
Boland et al [43]	*Infected pancreatic necrosis *Organ failure/sepsis	Open deb 9 (43%) Open CG 6 (29%) Lap deb 4 (19%) Lap CG 2(9%)	21 (100)	Nr	77 (32–155)	*Enterococcus* 8 Candida species 7 *E. Coli* 5	1 (open deb)	Nr	Nr	Nr	1 (open deb)	Nr	Nr	Nr
Raraty et al [11]	*Infected pancreatic necrosis FNA + 15 *Suspected Infected pancreatic necrosis/Organ failure/sepsis 13	transperitoneal necrosectomy -Midline17 -Transvers11	15 (53.6)	> 50% 31 (60%)	34 (5–149)	*E. coli*	6 (21.4)	median number of interventions 1 (1–9)	Nr	4	Nr	Nr	Nr	Nr
van Santvoort et al [13]	*Infected pancreatic necrosis FNA + *suspected Infected pancreatic necrosis/organ failure/sepsis	Surgical necrosectomies through bi-subcostal incision	42 (93)	< 30% 19 (42%) 30–50% 10 (22%) > 50% 16 (36%)	29 (12–155)	Nr	7 (16)	14	19 (42)	17 (38)	10 (22)	17 (38)	15 (33)	6

*Ho* homogeneous, *He* heterogeneous, *IPN* infected pancreatic necrosis, *CV* cardiovascular disease, *P* pulmonary disease, *CRI* chronic renal disease, *D* diabetes, *OT* other, *PF* pancreatic fistula, *POB* postoperative bleeding, *NB* new-onset diabetes, *PEI* pancreatic exocrine insufficiency

As in the inclusion criteria, all studies include patients submitted to primary ON for suspected or confirmed IPN but, in the same studies, also organ failure, prolonged pain, bleeding, persisting unwellness, and/or gastric outlet obstruction are considered as indications for open surgery. The reported time until necrosectomy from admission in days includes great excursions and ranges from 0 to 155 days.

Most studies describe results on ON with the use of longitudinal midline or bilateral subcostal trans-peritoneal approach. Only three studies reporting the use of different techniques, of which at least one is the traditional open approach, have been added to the review. Nine out of twenty studies involved in the review report data on the necessity of re-necrosectomy. On a total of 879 patients, a positive bacteriological culture was described in 740, confirming the preoperative suspicion of IPN in 84.2% cases. The most frequently pathogens found are *E. coli*, *Klebsiella*, *Acinetobacter*, *Pseudomonas*, *Enterococcus*, and *Candida species.*

Mortality rate was reported in all studies with a high variability ranging from 6.7 to 64.1, while not all studies are complete in describing acute or chronic post-operative complications. Indeed, seven studies added information on new onset of post-operative organ failure with 108 patients (34.6%) on 312 of the reporting studies developing new organ failure. Acute post-operative complications have been pointed out in seventeen studies with 49.4% of patients (385 on 779) developing pancreatic fistula or post-operative bleeding. Chronic complications are debated in six studies showing, on a pool data of 230 patients, the new onset of diabetes in 52 patients (22.6%) and pancreatic exocrine insufficiency in 29 (12.6%).

## Discussion

The revised version of the Atlanta Classification in 2012 has replaced the nomenclatures of 1992 for various types of acute pancreatitis complications, such as pancreatic abscess, initially defined as a “localised collection of purulent material without significant necrotic material.” It has allowed to make a clear distinction in different pancreatic collections [45]. The collections with only fluid content usually occur from interstitial oedematous pancreatitis and are defined as acute peripancreatic fluid collection if these occurs during the first weeks from the arising of the pancreatitis, or pancreatic pseudocyst as a delayed (usually > 4 weeks) complication. On the other hand, collections arising from ANP usually contain a solid and fluid component. These are defined as acute necrotic collection occurring in the early phase of pancreatitis and before demarcation, or WON, which represents a heterogeneous spectrum of solid and liquid collections (necrotic debris and fluid) surrounded by a radiologically identifiable capsule (which rarely develops before 4 weeks from onset of pancreatitis) [3].

Infection of such pancreatic fluid or necrotic collection is an unusual circumstance reported in literature, occurring in approximately 30% of patients with necrotizing pancreatitis but is related to a high mortality rate (20%) [8–14]. The common clinical manifestations of IPN are persistent fever, abdominal discomfort, or back pain accompanied to early satiety, anorexia, weight loss, abdominal distention, vomiting, or worsening reflux due to partial or complete gastric or duodenal outlet obstruction [46, 47]. The presence of a systemic inflammatory response syndrome, with modification of inflammatory markers (elevation of C-reactive protein, progressive leucocytosis or positive blood cultures) and the onset of clinical deterioration in a stable patient on adequate support, with new or prolonged organ failure, are suggestive for IPN in 80% of cases [46, 47]. Jaundice may also occur secondary to biliary obstruction while symptoms related to hemorrhage into WON and gastroduodenal and/or splenic arterial erosions are more critic with hemodynamic instability.

Imaging signs such as gas in peripancreatic collections with or without a rapid accumulation on serial imaging are accurate predictors of IPN in the majority of patients [48–50]. The diagnosis of IPN, indeed, can be suspected by the patient’s clinical course, by the presence of gas within the heterogeneous or homogeneous collection seen on contrast enhancement CT or when percutaneous, image-guided, fine-needle aspiration (FNA) is positive for bacteria and/or fungi on Gram stain and culture [3, 47]. Some studies on small series of patients with IPN confirmed the results of our systematic review showing that the pathogens usually recognized on bacterial culture were oral commensal bacteria or highly virulent enteric bacteria such as *Escherichia coli*, *Pseudomonas aeruginosa*, *Klebsiella*, or *Enterococcus spp*, and that, less commonly, patients were found to be infected with multiple pathogens [51].

The radiological findings evocating infection of necrotic collection or bacterial positivity on FNA, as in our series, always represent an indication for intervention [9].

The ON has been originally described by Beger et al., and it has represented, for a long time, the gold standard for the treatment of necrotizing pancreatitis with secondary infection. It consists in a laparotomy through a bilateral subcostal or longitudinal incision, removal of all necrotic tissue, drains insertion, and abdomen closure [52]. Despite its employment for selected cases, in the clinical practice, as demonstrated by this review, this approach is still characterized by high rates of complications (34 to 95%) and death (11 to 39%) and with a risk of long-term pancreatic insufficiency [14]. Husu et al. [27] declare that mortality after ON depends on patient’s preoperative risk factors. According to their experience, patients with an average age over 60 years, with preoperative significant comorbidities, necrosectomy within 4 weeks from the clinical onset, and deterioration or prolonged organ failure, apparently seem to report a higher mortality rate. The association of organ failure and IPN with higher mortality rate has also been previously highlighted by Petrov et al. [53], who claimed that the relative risk of mortality doubles when organ failure and IPN are both present. This statement is confirmed by this literature review. Indeed, Pascual et al. [38] reported the association between the incidence of perioperative single or multi-organ failure (single 14.3%; multiple 52.4%) and pre-existing significant comorbidities (52.4%) with a high mortality rate (42.9% of total cohort’s study). Also, Šileikis et al. [30] reported a higher mortality rate (64.1%) associated with perioperative multi-organ failure rate of 77.3%. Some studies have showed how mortality rate could be negatively influenced by the surgical management within 4 weeks or the low-range time. Indeed, Bausch et al. [41] reported a mortality rate of 63.3% for patients submitted to ON with a low average time of 11 days (ranging from 0 to 77). On the other hand, Senthil Kumar et al. [40] report a mortality rate of 6.7% on a cohort of 15 patients submitted to primary ON for IPN (100% post-operative positive bacterial culture) with an average time from the onset of malaise and surgery of 31 days (range 17–45).

In the last decade, the growing consent for minimal invasive procedures, also in pancreatic disease, has provided valid alternative strategies to the open approach [8–16]. Less invasive techniques, including ETN, PD, and minimally invasive necrosectomy, are increasingly being used with favourable results.

In 2010, Van Santvoort et al. [14], in their multicentre RCT, showed that the preferred treatment strategy for patients with necrotizing pancreatitis and secondary infection, both from a clinical and an economic point of view, may be considered a minimally invasive step-up approach (PD or endoscopic drainage of the collection) followed, if necessary, by minimally invasive VARD.

This interventional strategy, requiring always a multidisciplinary approach, has been largely discussed in the recent literature. Subsequently, a lot of studies and some RCT trials have systematically compared the endoscopic approach to surgical step-up approach with percutaneous and mini-invasive necrosectomy and/or ON, in order to assess if these minimally invasive approaches were associated with better outcomes in terms of complications, mortality, and length of stay. The PENGUIN trial [54] has compared the endoscopic step-up approach with surgical step-up approach consisting in VARD or laparotomic ones and has showed that the ETN reduced the proinflammatory response and new onset organ failure. The TENSION trial [55] has compared the endoscopic step-up approach (endoscopic ultrasound-guided transluminal drainage followed, if necessary, by endoscopic necrosectomy) with the surgical step-up approach (PD followed, if necessary, by VARD). Despite a not clear superiority of the endoscopic group on the major complication and mortality, they have concluded that the endoscopic step-up approach is probably the treatment to prefer in consideration of the better results for the minor outcomes. Recently, in the MISER trial [56], the outcomes of minimally invasive surgery (laparoscopic or VARD, depending on the location of collection) vs endoscopic transluminal approaches for patients with IPN have been compared showing that the endoscopic approach for IPN significantly reduced major complications, lowered costs, and increased quality of life.

Nevertheless, all these studies are difficult to compare for the variability of patient characteristics and differences between surgical interventions. Indeed, Haney et al. [57] have highlighted differences concerning also drainage techniques, placement of nasocystic catheters, and irrigation of the cyst in the endoscopic procedures among different trials. Moreover, these recommendations do not take into account the large variability in expertise between centres with various techniques but highlighted the importance of an expert pool involvement of specialists composed of gastroenterologist, interventional radiologists, surgeons, and intensive care physicians that may improve the patient outcomes. The whole knowledge of a severe pancreatic disease, usually characterized by severe clinical conditions and hemodynamic instability, allows to perform a correct clinical evaluation and to determine the complete therapeutic approach, also defining the suitable timing for treatment and, eventually, the type of interventional options [8].

## Conclusions

Infected pancreatic necrosis results to be a difficult and challenging disease, hazardous to manage, and often taking advantage in a multidisciplinary approach. In a scientific community who actually prefers the employment of mini-invasive strategies, open necrosectomy is confirmed to be the last resort, useful in selected severe cases, with a defined timing and in case of proven non-feasibility and no advantage of other minimally invasive approach.

## Data Availability

All data generated or analyzed during this study are included in this published article.

## Abbreviations

AP: Acute pancreatitis
ANP: Acute necrotic pancreatitis
WON: Walled-off necrosis
IPN: Infected pancreatic necrosis
ON: Open necrosectomy
PD: Percutaneous drainage
ETN: Endoscopic transgastric necrosectomy
VARD: video-assisted retroperitoneal debridement
RCT: Randomized controlled trials
CT: Computed tomography
CRP: C-reactive protein
MRCP: Magnetic resonance cholangiopancreatography
BD: Bile duct
ERCP: Endoscopic retrograde cholangiopancreatography
US: Ultrasonography
OGD: Oesophagogastroduodenoscopy
CTI: CT: Severity index
APACHE II: Acute Physiologic and Chronic Health Evaluation II
ICU: Intensive care unit
ASA: American Society of Anaesthesiologists
FNA: Fine-needle aspiration
